# Supplementary material for: Genome-Wide Identification and Expression Analysis of AMT and NRT Gene Family in Pecan (Carya illinoinensis) Seedlings Revealed a Preference for NH4+-N
Source: Int J Mol Sci. 2022 Nov 1;23(21):13314. doi: 10.3390/ijms232113314 (PMC9655437; doi:10.3390/ijms232113314)

Figure S1. Synteny analysis of *AMT* and *NRT* gene families in pecan.

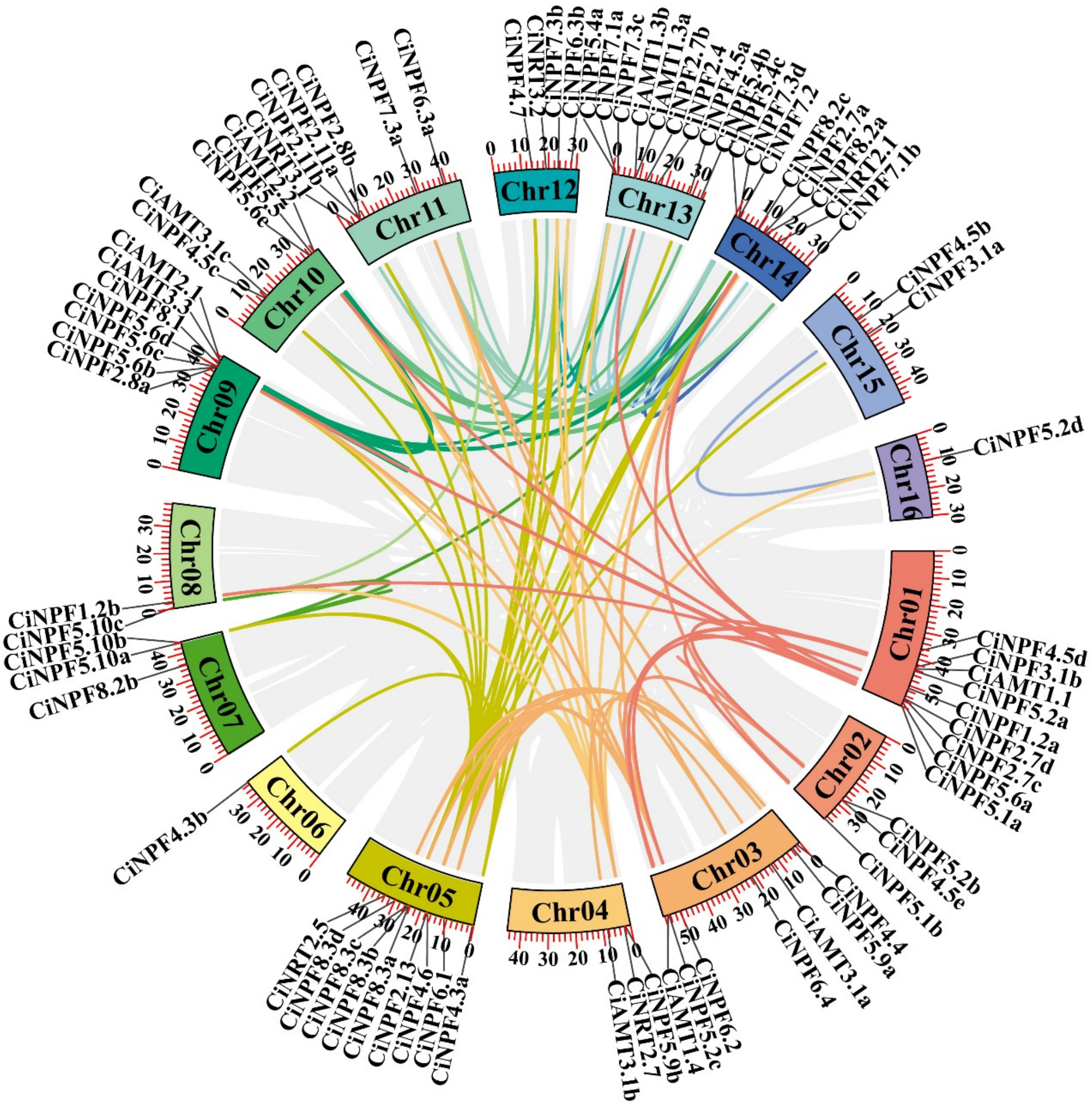

Figure S2. Cis-elements analysis of *AMT* genes in pecan (*Carya illinoinensis*). ABRE involved in the abscisic acid responsiveness; ARE involved in anaerobic responsiveness; CAAT-box involved in promoter and enhancer regions; TATA-box involved in core promoter element around -30 of transcription start; Light Responsive involved in light responsiveness; LTR involved in low-temperature responsiveness; MeJA responsive involved in the MeJA-responsiveness, include CGTCA-motif and TGACG-motif; TCA-element involved in salicylic acid responsiveness; TGA-element involved in auxin responsiveness; TC-rich repeats involved in defense and stress responsive; GC-motif involved in anoxic specific inducibility; Circadian involved in circadian control; MBS involved in drought-inducibility.

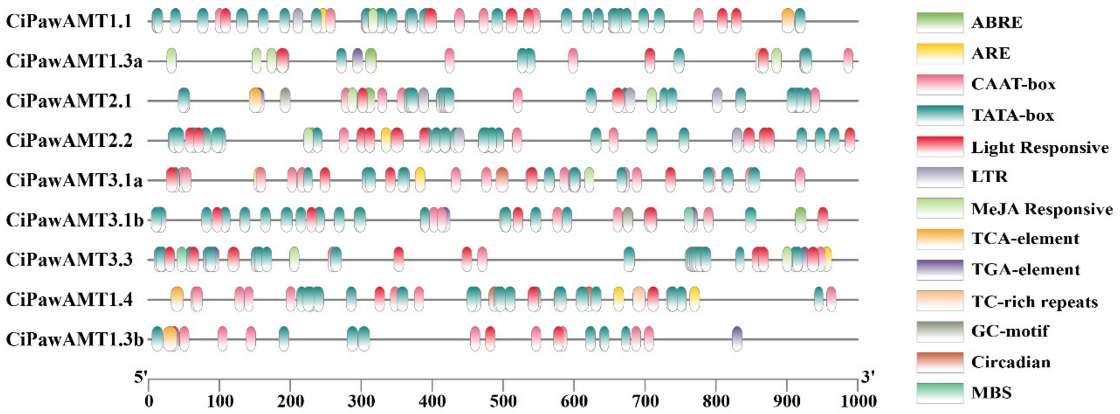

Figure S3. Cis-elements analysis of *NRT* genes in pecan (*Carya illinoinensis*). ABRE involved in the abscisic acid responsiveness; ARE involved in anaerobic responsiveness; TATA-box involved in core promoter element around -30 of transcription start; MBS involved in drought-inducibility; Light Responsive involved in light responsiveness; CAT-box involved in meristem expression; CAAT-box involved in promoter and enhancer regions; MeJA responsive involved in the MeJA-responsiveness, include CGTCA-motif and TGACG-motif; RY-element involved in seed-specific regulation; TGA-element involved in auxin responsiveness; GCN4_motif involved in endosperm expression; TCA-element involved in salicylic acid responsiveness; LTR involved in low-temperature responsiveness; GC-motif involved in anoxic specific inducibility; Circadian involved in circadian control; TATC-box involved in gibberellin-responsiveness; TC-rich repeats involved in defense and stress responsive; HD-Zip 1 involved in differentiation of the palisade mesophyll cells; MBSI involved in flavonoid biosynthesis.

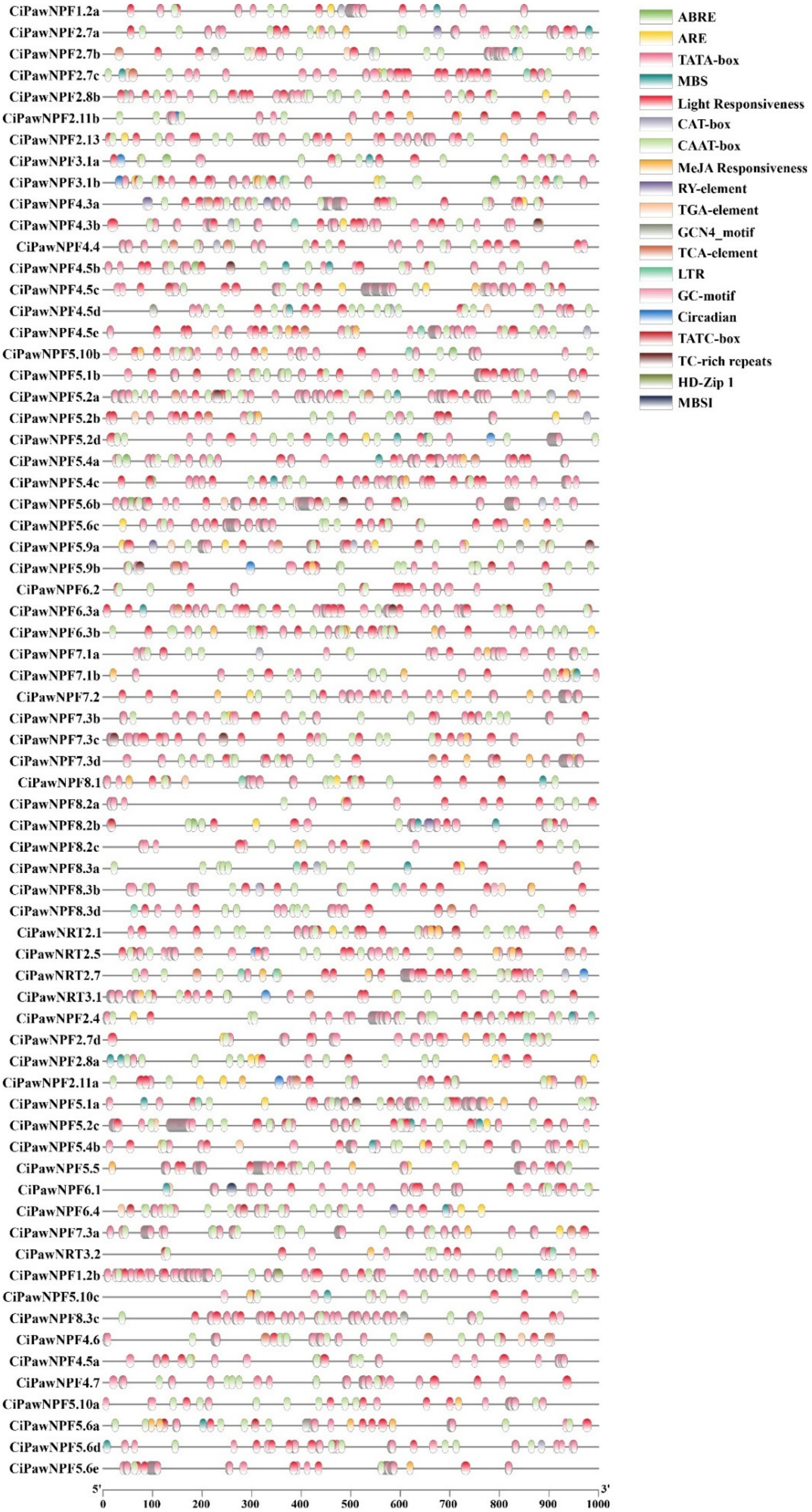

Figure S4. Protein-protein interaction network of *AMT* and *NRT* genes in *Arabidopsis*.

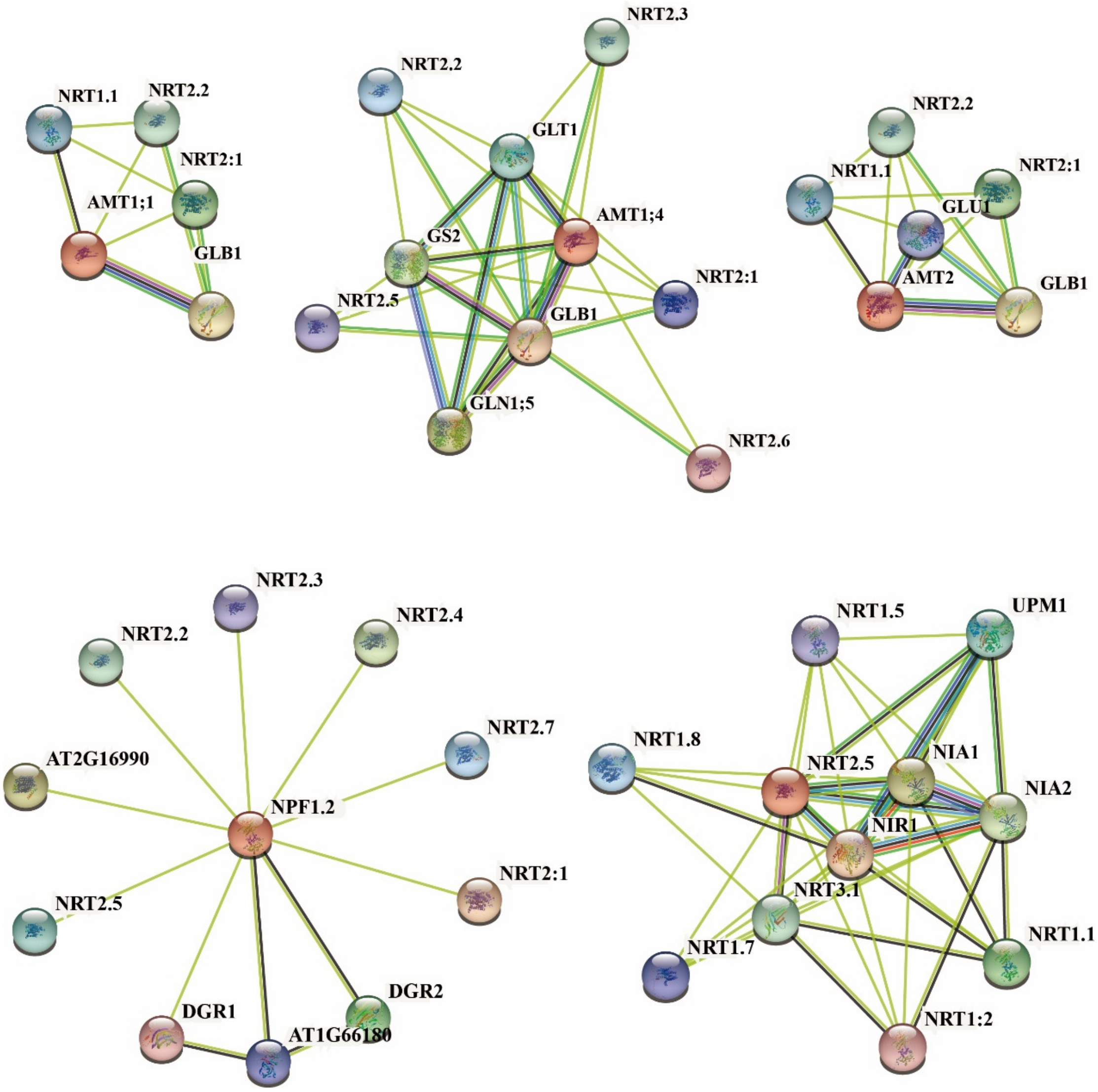

Supplement: Supplementary file 1 [file ijms-23-13314-s001.zip › Supplementary figures.pdf]
